# Decreased phosphatase PTEN amplifies PI3K signaling and enhances proinflammatory cytokine release in COPD

**DOI:** 10.1152/ajplung.00382.2016

**Published:** 2017-05-18

**Authors:** Satoru Yanagisawa, Jonathan R. Baker, Chaitanya Vuppusetty, Peter Fenwick, Louise E. Donnelly, Kazuhiro Ito, Peter J. Barnes

**Affiliations:** Airway Disease Section, National Heart and Lung Institute, Imperial College London, London, United Kingdom

**Keywords:** chronic obstructive pulmonary disease, phosphatase and tensin homolog deleted from chromosome 10, PI3K IL-6, oxidative stress

## Abstract

The phosphatidylinositol 3-kinase (PI3K) pathway is activated in chronic obstructive pulmonary disease (COPD), but the regulatory mechanisms for this pathway are yet to be elucidated. The aim of this study was to determine the expression and role of phosphatase and tensin homolog deleted from chromosome 10 (PTEN), a negative regulator of the PI3K pathway, in COPD. PTEN protein expression was measured in the peripheral lung of COPD patients compared with smoking and nonsmoking controls. The direct influence of cigarette smoke extract (CSE) on PTEN expression was assessed using primary lung epithelial cells and a cell line (BEAS-2B) in the presence or absence of l-buthionine-sulfoximine (BSO) to deplete intracellular glutathione. The impact of PTEN knockdown by RNA interference on cytokine production was also examined. In peripheral lung, PTEN protein was significantly decreased in patients with COPD compared with the subjects without COPD (*P* < 0.001) and positively correlated with the severity of airflow obstruction (forced expiratory volume in 1-s percent predicted; *r* = 0.50; *P* = 0.0012). Conversely, phosphorylated Akt, as a marker of PI3K activation, showed a negative correlation with PTEN protein levels (*r* = −0.41; *P* = 0.0042). In both primary bronchial epithelial cells and BEAS-2B cells, CSE decreased PTEN protein, which was reversed by *N*-acetyl cysteine treatment. PTEN knockdown potentiated Akt phosphorylation and enhanced production of proinflammatory cytokines, such as IL-6, CXCL8, CCL2, and CCL5. In conclusion, oxidative stress reduces PTEN protein levels, which may result in increased PI3K signaling and amplification of inflammation in COPD.

chronic obstructive pulmonary disease (COPD) is associated with amplified inflammatory responses, predominantly in small airways and lung parenchyma (16, 18). Long-term inhalation of noxious gases such as cigarette smoke are the main causal mechanism for this persistent inflammation (31, 36, 38, 43, 48). Although a multiplicity of cells and mediators are involved in the pathophysiology of COPD (7), many reports have suggested the importance of phosphatidylinositol 3-kinase (PI3K) and its downstream target Akt, both of which are strongly upregulated by the oxidants (5, 20, 34a). Recently, we have shown that the PI3K is significantly activated in peripheral blood mononuclear cells and associated with the COPD disease progression, partly through corticosteroid resistance (44). These results suggest that the oxidant-induced enhancement of the PI3K pathway has been altered in patients with COPD and may contribute to its persistent inflammation. In addition, PI3K signaling (determined by Akt phosphorylation) was reported to be more activated in airway epithelial cells collected from COPD subjects when compared with those from healthy subjects (15). However, the regulatory mechanisms for prolonged activation of PI3K and its downstream signaling pathway have not yet been determined.

Phosphatase and tensin homolog deleted from chromosome 10 (PTEN) is a well-described negative regulator of PI3K, which converts phosphatidylinositol-3,4,5-phosphate (PIP3) to phosphatidylinositol-4,5-phosphate (PIP2), leading to the inactivation of downstream target Akt and/or phosphoinositide-dependent kinase (PDK)1 (41). PTEN was originally discovered as a tumor suppressor gene that encodes a phosphatase involved in inactivating a growth and differentiation (27, 42) and was reported to be frequently mutated or deleted in the epithelium of smokers and in lung cancer (22). However, only limited research has been conducted to elucidate the role of PTEN in the pathogenesis of COPD, despite the fact that COPD is another important disease associated with cigarette smoking. Single nucleotide polymorphism analysis has demonstrated that PTEN polymorphism is an important risk factor for COPD (19). In addition, in vitro data have demonstrated that cigarette smoke extract (CSE) reduces PTEN protein levels (40) and that oxidative stress impairs its activity (4). Therefore, the present study was designed to clarify whether PTEN in lung is downregulated in patients with COPD. In addition, we also investigated whether reduced PTEN protein by oxidative stress in bronchial epithelial cells was linked to the secretion of proinflammatory cytokines that are relevant to the pathophysiology of COPD.

## MATERIALS AND METHODS

### 

#### Reagents.

Commercially available reagents were obtained as follows: RPMI medium 1640 (RPMI 1640) (Cat. No. 11875), silencer select negative control no. 1 short interference (si) RNA (Cat. No. 4390844) as transfection negative control for knockdown (KD) experiments, Lipofectamin RNAiMAX (Cat. No. 13778), polymerase chain reaction (PCR) primers for PTEN (Hs00829813), GNB2L1 (Hs00272002), interleukin (IL)-6 (Hs00174131), CXCL8 (Hs00174103), matrix metalloproteinase-9 (MMP-9; HSs00234579), Muc5ac (HsS00873651), Muc5b (Hs00861595), tranforming growth factor-β (TGF-β; Hs00998133) were from Life Technologies (Carlsbad, CA); fetal bovine serum (FBS), *S*-nitroso-*N*-acetyl-dl-penicillamine (N3398), complete protease inhibitor cocktail (11836153001), thiazolyl blue tetrazololium bromide for MTT assay (M2003), l-buthionine-sulfoximine (BSO), MG-132 (Z-Leu-Leu-Leu-CHO) (C2211), hydrogen peroxide (H_2_O_2_) (H1009), *N*-acetyl cysteine (A9165), and dexamethasone (D1756) were from Sigma-Aldrich (St. Louis, MO); rabbit-derived anti-PTEN antibody (ab154812) and anti-β-actin antibody (ab6276) were from Abcam (Cambridge, UK); anti-phosphorylated-Akt (Ser47) (p-Akt) antibody (Cat. No. 9271), anti-total Akt antibody (Cat. No. 4691), and PTEN siRNA (Cat. No. 6538) were from Cell Signaling Technology (Danvers, MA); anti-Nrf2 antibody (sc-13032) was from Santa Cruz Biotechnology (Santa Cruz, CA); ALLN (*N*-acetyl-Leu-Leu-Norleu-al; Cat. No. 208750) was from EMD Millipore (Billerica, MA); recombinant human IL-1β was R&D Systems (Minneapolis, MN); and goat-derived peroxidase-conjugated anti-mouse (P0447) or anti-rabbit (P0448) secondary antibodies were from Dako (Cambridge, UK).

#### Peripheral lung tissue.

COPD patients were categorized according to Global Initiative for Chronic Obstructive Lung Disease (GOLD) stage (35). Peripheral lung tissues from subjects with normal lung function [nonsmokers (NS); 8 subjects], smokers without COPD (SM; 9 subjects), and 26 patients with mild to very severe COPD (stage 1, 9 subjects; stage 2, 8 subjects; stage 3, 3 subjects; and stage 4, 6 subjects) were obtained using a tissue bank linked to an established patient registry from the patients who have made a decision to proceed with lung resection for the treatment of a lung tumor (12), and protein extracts were prepared using RIPA buffer (Sigma-Aldrich; 150 mM NaCl, 1.0% IGEPAL CA-630, 0.5% sodium deoxycholate, 0.1% sodium dodecyl sulfate-polyacrylamide, and 50 mM Tris·HCl, pH 8.0) completed with protease inhibitor as previously described (21). We analyzed PTEN protein expression in a blinded manner by Western blot analysis.

#### Immunoprecipitation and PTEN activity assay.

The protein extract of peripheral lung tissues was immunoprecipitated using an anti-PTEN antibody, and its phosphatase activity was measured by a *p*-nitrophenyl phosphatase (pNPP) protein phosphatase assay kit (AS-71105; AnaSpec, Fremont, CA) following the manufacturer’s instructions. Briefly, 200 μg/200 μl of protein extracts were reacted on a shaker platform with 8 μl of rabbit-derived anti-PTEN antibody (Cat. No. 9188; Cell Signaling Technology) overnight. Then, 30 μl of protein A magnetic beads (Cat. No. 88845; Thermo Fisher Scientific Pierce Biotechnology, Rockford, IL) were added and incubated on the shaker at 4°C for 4 h. After being washed four times with 100 mM Tris·HCl (pH 8.0), the PTEN combined beads were dissolved in a total 100 μl of PTEN reaction buffer (100 mM Tris·HCl pH 8.0 and 10 mM dithiothreitol) (30) complemented with pNPP solution, and incubated at 37°C for the appropriate duration. Then the supernatant was transferred to a 96-well microplate, and optical densities (OD) measured at a wavelength of 405λ. The results of pNPP assay were calculated by subtracting the OD_405_ value of negative control from OD_405_ of each sample. In addition, the PTEN proteins were eluted from the beads by boiling, separated by SDS-PAGE and detected by Western blot analysis with mouse-derived anti-PTEN antibody (sc-7974: Santa Cruz Biotechnology). Finally, PTEN activity was normalized by PTEN protein level (PTEN activity/protein).

#### Cell culture and stimulation.

Human primary bronchial epithelial cells were cultured as monolayers in LHC-9 media (Invitrogen, Paisley, UK) on collagen (1% wt/vol)-coated plates. Cells were extracted from lung tissue from patients undergoing lung resection surgery at the Royal Brompton Hospital. All subjects gave informed written consent, and the study was approved by the National Research Ethics Service London-Chelsea Research Ethics Committee (Study No. 09/H0801/85). The BEAS-2B cell line (SV40-immortalized human airway bronchial epithelial cell line) were purchased from the American Culture of Tissue Collection and were maintained in complete growth medium (RPMI 1640 supplemented with heat-inactivated 10% FBS and 1% l-glutamine) at 37°C/5% CO_2_. Before use, cells were starved for 24 h in minimum medium (RPMI 1640 supplemented with 1% FBS and 1% l-glutamine). *N*-acetyl cysteine (10 mM) was also given 10 min before CSE treatment.

#### Preparation of CSE.

One full-strength Marlboro cigarette with the filter removed (Phillip Morris, London) was bubbled into 10 ml of minimum medium, at a rate of one cigarette per 1.5 min. CSE was then passed through a 0.2-μm filter to sterilize and remove particulate matter and was used immediately. The optical density was measured at 320λ wavelength, and the solution was diluted to be OD = 0.85 (this is original stock as 100%). The stock CSE was thereafter diluted with culture media to appropriate percentages of CSE solution.

#### RNA interference.

BEAS-2B cells were transfected with PTEN siRNA (100 nM) or random oligonucleotide control (100 nM) for 48 h using Lipofectamin RNAiMAX, according to the manufacturer’s instructions.

#### Western blot analysis.

After stimulation, whole cell extracts were prepared using RIPA buffer (19), separated by SDS-PAGE, transferred to nitrocellulose membrane, and then incubated with anti-PTEN antibody (1:1,000 dilution), anti-phosphorylated-Akt (p-Akt) antibody (1:500 dilution), anti-total-Akt antibody (1:1,000 dilution), or anti-Nrf2 antibody (1:1,000 dilution) overnight. To standardize the expression of each protein, the membranes were reprobed with anti-β-actin antibody (1:200,000 dilution). The membranes were then incubated with the appropriate peroxidase-conjugated secondary antibodies (1:3,000 dilution, each). The bound antibodies were visualized by chemiluminescence (ECL plus; GE Healthcare, Buckingham, UK).

#### RT-quantitative PCR.

Total cellular RNA was extracted using RNeasy mini kit (Qiagen, Valencia, CA), and cDNA was prepared by using Multiscribe reverse transcriptase (Applied Biosystems, Warrington, UK). The RT-qPCR analysis of PTEN, IL-6, CXCL8, MUC5AC, MUC5B, MMP-9, TGF-β, and GNB2L1 as a housekeeping gene was performed using Taqman primers and probe set from Applied Biosystems in a Corbett Rotor-Gene 3000 (Corbett Research Sortlake, Sydney, Australia).

#### Cytokine ELISA assay.

Cytokine concentrations in the cell supernatant was assessed by human cytokine array panel A (ARY005: R&D Systems), and concentrations of IL-6, CXCL8, CCL2, and CCL5 were determined by a human sandwich ELISA-kit (R&D Systems) according to the manufacturer’s instructions.

#### Statistical analysis.

Data from clinical samples were expressed as mean values ± SD. For the analysis of PTEN, statistical significance was assessed by Mann-Whitney *U*-test for single comparisons, or by using nonparametric Kruskal-Wallis test with appropriate post hoc analysis (Dunnett’s test) to exclude possible interaction between various variables within subgroups (Statcel 2; OMS Publishing, Saitama, Japan). The analysis of correlation between each factors was performed by Spearman’s correlation coefficient rank test. The in vitro data using BEAS-2B cells are expressed as mean values ± SE. Data were analyzed by one-way ANOVA followed by Tukey’s or Scheffé’s *F*-test to adjust for multiple comparisons. For *N*-acetyl cysteine treatment study, data were analyzed by one-way repeated ANOVA followed by Dunnett’s multiple comparison test. If the parametric analysis was not applicable, a Friedman test followed by Dunnett’s multiple comparison was performed. An unpaired two-tailed Student’s *t*-test was used for single comparisons. All reported *P* values are two sided, and *P* < 0.05 was considered statistically significant.

## RESULTS

### 

#### PTEN expression in peripheral lung and epithelial cells from COPD patients.

**Fig. 1.**
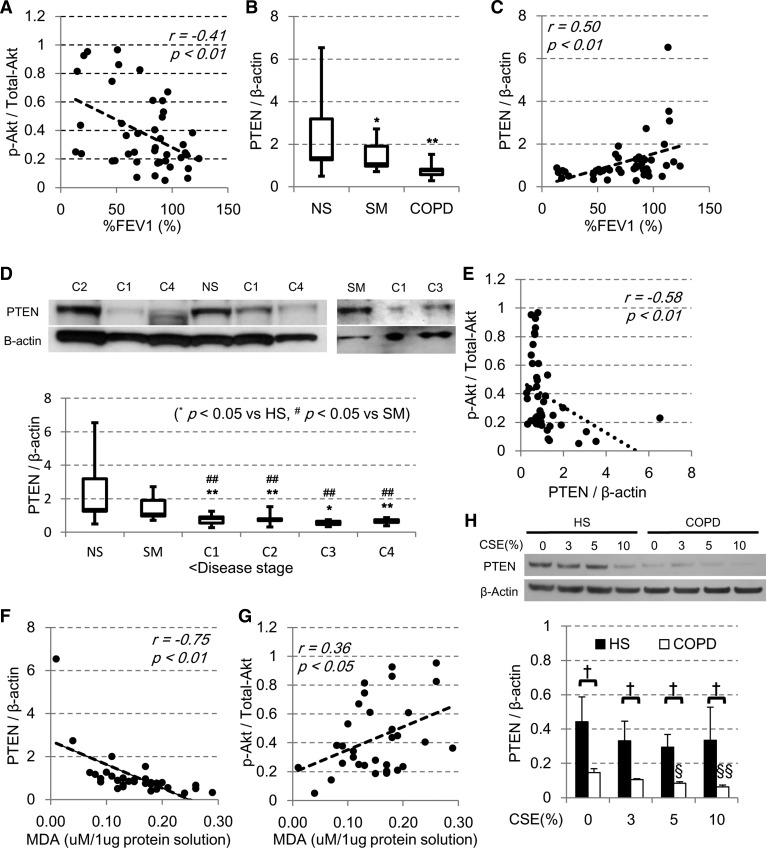
Phosphatase and tensin homolog deleted from chromosome 10 (PTEN) expression in peripheral lung and bronchial epithelial cells from chronic obstructive pulmonary disease (COPD). Whole tissue extracts were prepared from peripheral lung tissue of nonsmokers (NS; *n* = 8), smokers without COPD (SM; *n* = 9), patients with COPD stage 1 (C1; *n* = 9), COPD stage 2 (C2; *n* = 8), COPD stage 3 (C3; *n* = 3), and COPD stage 4 (C4; *n* = 6) in lysis buffer, and phosphorylated-Akt (p-Akt), total-Akt, PTEN, and β-actin protein levels were determined by Western blot analysis. *A*: correlation between the forced expiratory volume in 1-s percent predicted (%FEV_1_) and phosphorylated fraction of Akt (p-Akt/total-Akt). *B*: PTEN protein level in lung sample from healthy nonsmoker subjects (NS) and smokers without COPD (SM) and COPD patients (C1–4). *C*: correlation of PTEN protein levels and the %FEV_1_. *D*: representative Western blot image and PTEN protein levels of healthy subjects (NS, SM) and patients with COPD of each stage C1–4. *E*: correlation of PTEN protein levels and p-Akt/total-Akt. Correlation of malondialdehyde (MDA) with PTEN protein levels (*F*) and p-Akt/total-Akt (*G*). *H*: primary bronchial epithelial cells were isolated from the healthy subjects (HS; *n* = 4) or COPD patients (*n* = 4), and PTEN protein levels in the presence or absence of cigarette smoke extract (CSE) were examined by Western blot analysis. The effect of different concentrations of CSE on PTEN protein in BEAS-2B cells incubated for 24 h is shown. *P < 0.05, **P < 0.01, compared with the values of NS. ^§^*P* < 0.05, ^§§^*P* < 0.01, compared with the each CSE 0% group. ^†^*P* < 0.05, between the 2 groups.

The characteristics of the subjects are shown in Table 1. We first confirmed the activation status of the PI3K pathway by phosphorylation of its downstream kinase Akt by Western blot analysis. The phosphorylated fraction of Akt normalized to total Akt protein levels (p-Akt-to-total-Akt ratio) was significantly increased in COPD lung and was negatively correlated with the forced expiratory volume in 1-s percent predicted (%FEV_1_) (*r* = −0.41; *P* < 0.01; Fig. 1*A*), as we have previously reported (44). In these samples, the PTEN protein levels were significantly decreased in all patients with COPD, compared with those of the nonsmoking subjects with normal lung function (NS). The levels of PTEN protein in smokers without COPD (SM) was also significantly reduced compared with NS (*P* < 0.05; Fig. 1*B*). In addition, there was a significant positive correlation between the PTEN protein levels and airway obstruction measured by FEV_1_/FVC ratio (*r* = 0.65, *P* < 0.001) or the severity of air flow limitation by %FEV_1_ (*r* = 0.50; *P* < 0.01; Fig. 1*C*), indicating that the PTEN levels were decreased significantly as COPD progresses (Fig. 1*D*). Furthermore, the level of PTEN protein was negatively correlated with phosphorylated fraction of Akt (p-Akt/total-Akt) (*r* = −0.58; *P* < 0.01; Fig. 1*E*), which suggested the importance of PTEN in regulating the Akt phosphorylation in vivo. Despite the long smoking history of COPD patients, there was no apparent relationship between the PTEN protein levels and smoking status (*r* = −0.22; *P* = 0.16) or with age (*r *= −0.12; *P* = 0.42) or sex difference (*P* = 0.81). Interestingly, there was a negative correlation between PTEN protein levels and the level of malondialdehyde (MDA), an oxidative stress marker (*r* = −0.75, *P* < 0.01, Fig. 1*F*). MDA was also correlated with p-Akt/total-Akt ratio (*r* = 0.36, *P* < 0.05) (Fig. 1*G*). In addition, we also determined mRNA levels of PTEN in COPD lung samples. There was a trend of the reduction of mRNA of PTEN normalized to a housekeeping gene GNB2L1 but not statistically significant (NS: 0.25 ± 0.063, SM: 0.22 ± 0.056, GOLD 1/2: 0.22 ± 0.048, and GOLD 3/4: 0.11 ± 0.021).

**Table 1.** The characteristics of study subjects for peripheral lung tissues

	Nonsmokers	Smokers without COPD	COPD1	COPD2	COPD3	COPD4
*n*, men/women	8 (4/4)	9 (3/6)	9 (5/4)	8 (3/5)	3 (2/1)	6 (3/3)
Age, yr	56.6 ± 21.2	64.7 ± 12.7	69.2 ± 6.5	59.5 ± 7.2	63.3 ± 10.2	57.8 ± 4.4
Pack year	N/A	56.1 ± 34.1	53.0 ± 27.5	57.0 ± 37.8	46.0 ± 12.3	41.0 ± 16.7
FEV_1_, liter	3.02 ± 0.92	2.53 ± 0.62	2.48 ± 0.62	1.72 ± 0.41**^##^**	1.56 ± 0.55**^##^**	0.56 ± 0.14**^##^**
%FEV_1_, %	99.3 ± 18.2	97.0 ± 16.4	90.7 ± 7.5	60.3 ± 7.7**^##^**	46.9 ± 1.5**^##^**	18.1 ± 3.9**^##^**
FEV_1_/FVC ratio, %	81.8 ± 3.8	73.6 ± 2.1**^#^**	62.6 ± 5.3**^##^**	61.6 ± 9.0**^##^**	54.5 ± 6.9**^##^**	27.9 ± 7.5**^##^**
Inhaled CS	0/8	0/9	0/9	2/8	1/3	0/6
Systemic CS	0/8	1/9	0/9	1/8	0/3	0/6

Values are means ± SD. COPD patients were categorized by Global Initiative for Chronic Obstructive Lung Disease (GOLD) stage (33). COPD, chronic obstructive pulmonary disease; pack-year, (number of cigarettes smoked per day/20)_(pack)_ × duration of smoking_(year)_; FEV_1_, forced expiratory volume in one second; %FEV_1_ = FEV_1_, %predicted normal; FVC, forced vital capacity; CS, corticosteroids.

# *P* < 0.05 vs. nonsmokers.

## *P* < 0.01 vs. nonsmokers.

As the peripheral lung contains many different type of cells, we examined PTEN protein levels in primary human bronchial epithelial cells isolated from subjects with or without COPD (Table 2) in the presence or absence of oxidant exposure (CSE). As shown in Fig. 1*H*, COPD patients showed the decreased protein levels of PTEN at baseline, which was further reduced in the presence of CSE in a dose-dependent manner (Fig. 1*H*); therefore, PTEN protein expression was reduced in bronchial epithelial cells and the cells seemed to be an important target of exogenous oxidative stress with resultant PTEN reduction.

**Table 2.** The characteristics of study subjects for primary epithelial cells

	COPD
*n*, men/women	3/1
Age, yr	68.75 ± 9.8
Pack year	82 ± 58.7
FEV_1_, liter	1.56 ± 3.1
%FEV_1_, %	62 ± 18.2
FEV_1_/FVC ratio, %	49 ± 14

Values are means ± SD. COPD patients were categorized by GOLD stage (33).

#### PTEN activity in peripheral lung from COPD patients.

**Fig. 2.**
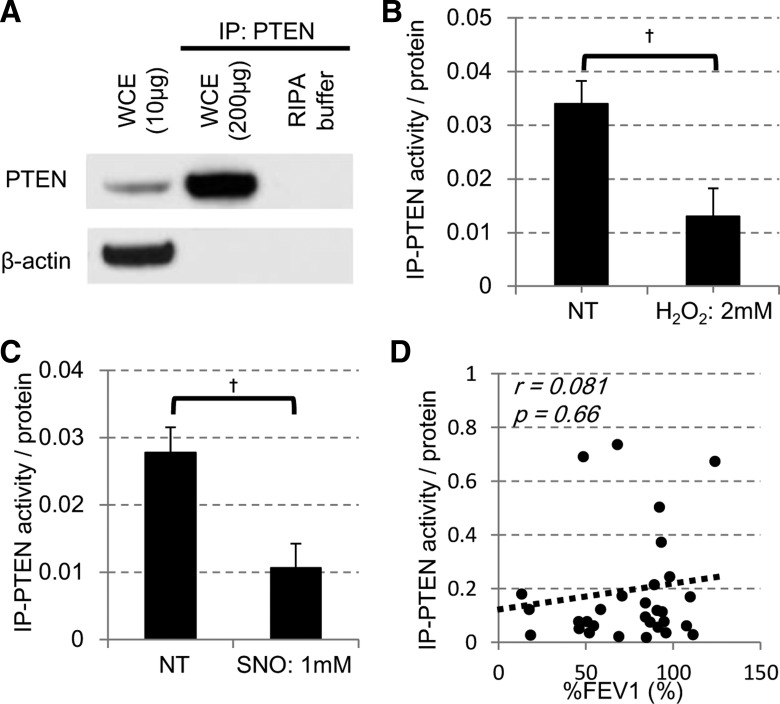
PTEN activity in BEAS2B cells and peripheral lung from COPD. *A*: protein extracts of BEAS-2B cells were immunoprecipitated for PTEN and detected by Western blot analysis. WCE, whole cell extracts. *B*: BEAS-2B cells were treated with or without [nontreated (NT)] 2 mM hydrogen peroxide (H_2_O_2_) for 24 h, and PTEN was immunopurified and IP-PTEN activity measured. *C*: IP-PTEN was directly treated with 1 mM *S*-nitrosothiol (SNO), and its activity was measured. †*P* < 0.05. *D*: correlation of PTEN activity levels and the %FEV_1_.

We also examined the levels of immunopurified (IP) PTEN activity (Fig. 2*A*) using the pNPP assay. The reliability of pNPP assay was first confirmed using samples from bronchial epithelial BEAS-2B cell line in vitro. As shown in Fig. 2*B*, IP-PTEN activity collected from BEAS2B cells treated with hydrogen peroxide (H_2_O_2_; 2 mM for 10 min at 37°C) was significantly reduced. The activity of IP-PTEN was also reduced by treatment of *S*-nitrosothiol (SNO; 1 mM for 10 min at 25°C; Fig. 1*C*). Thus, in BEAS2B cells, oxidative stress potentially reduced PTEN activity, as previously reported (23, 24, 52). In this assay system, we examined PTEN activity immunopurified from peripheral lung samples available. As the assay was underpowered, we did not find any difference of the IP-PTEN activities between COPD patients and non-COPD subjects. In addition, there was no significant correlation between the IP-PTEN activities determined in this condition and the FEV_1_/FVC (*P* = 0.63) nor %FEV_1_ (*P* = 0.66, Fig. 2*D*).

#### CSE-induced PTEN reduction in bronchial epithelial cells.

**Fig. 3.**
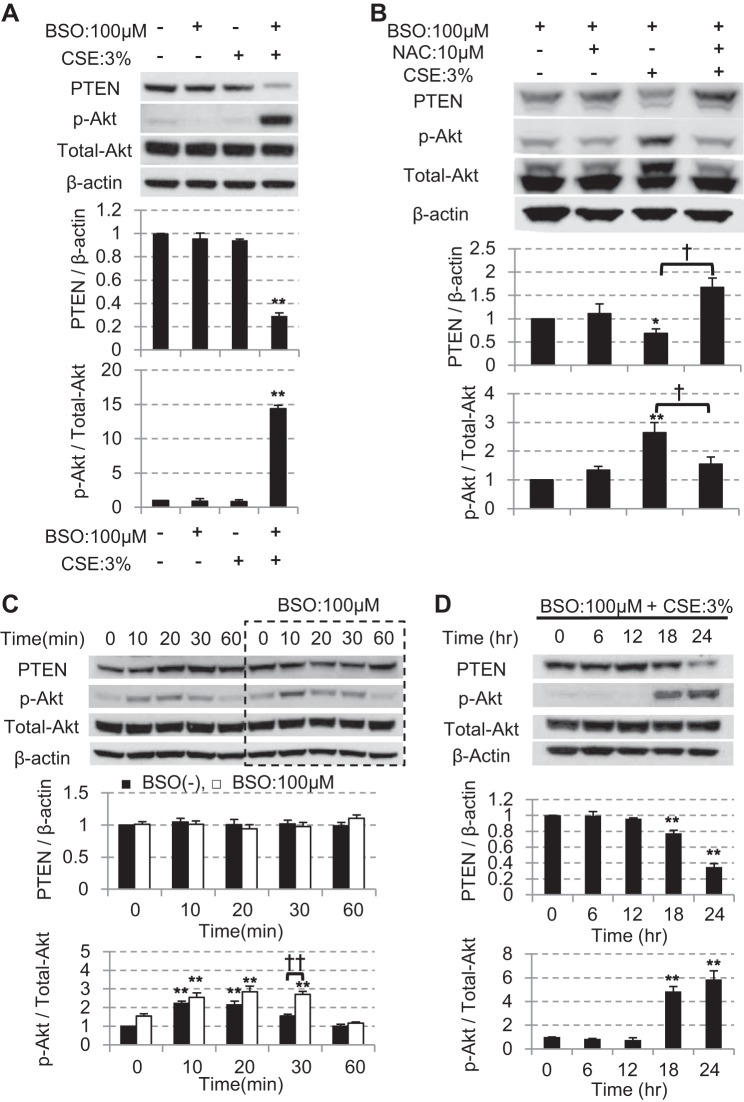
The effect of CSE on PTEN and PI3K signaling after short- and long-term exposure in BEAS-2B cells. *A*: after 16 h pretreatment with or without 100 μM l-buthionine-sulfoximine (BSO), BEAS-2B cells were cultured in the presence or absence of the 3% CSE for 24 h, and then PTEN protein expression (corrected with β-actin) and Akt phosphorylation (corrected with total Akt) were determined by Western blot analysis (*n* = 3). *B*: effect of *N*-acetyl cysteine (10 mM, 30 min before CSE stimulation) on CSE (3%) induced reduction of PTEN and elevation of Akt phosphorylation (*n* = 5). *C*: effect of 3% CSE on PTEN (*top*) or p-Akt/total-Akt (*bottom*) up to 60 min in the presence or absence of 100 μM BSO. *D*: time-dependent effect of 3% CSE on PTEN (*top*) and p-Akt/total-Akt (*bottom*) at 6, 12, 18 and 24 h after stimulation in the presence or absence of BSO pretreatment. All values are mean values ± SE of at least 3 experiments. *P < 0.05, **P < 0.01, compared with the values of nontreatment group. ^†^*P* < 0.05, ^††^*P* < 0.01, between the 2 groups.

On the basis of data from clinical samples, we explored the molecular mechanisms of PTEN reduction using BEAS-2B cells in vitro. When BEAS-2B cells are preincubated with l-buthionine-sulfoximine (BSO), an irreversible inhibitor of γ-glutamylcysteine synthetase that depletes the intracellular glutathione (13), the PTEN protein levels were significantly reduced after 24-h exposure with as low as 3% CSE in the presence of 100 μM BSO (Fig. 3*A*). Under these conditions, there was no reduction in cell viability (relative cell viability vs. nontreated cells: 0.90 ± 0.04, not statistically significant (data not shown). Conversely, Akt was significantly phosphorylated (Fig. 3*A*), as observed in clinical samples. The reduction of PTEN and an increase in Akt phosphorylation by 3% of CSE at 24 h were significantly inhibited by pretreatment of an anti-oxidant agent, *N*-acetyl cysteine (10 mM; Fig. 3*B*), suggesting the reduction of PTEN was oxidative stress dependent. In addition, the mechanisms for Akt phosphorylation seemed to differ between early and late phase after CSE exposure; although the acute Akt phosphorylation was transient and did not accompany the suppression of PTEN protein level (Fig. 3*C*), the Akt phosphorylation at later time points was associated with reduced PTEN expression (Fig. 3*D*). Thus PTEN reduction contributes to the prolonged phosphorylation of Akt even 24 h after CSE exposure (Fig. 3*D*).

**Fig. 4.**
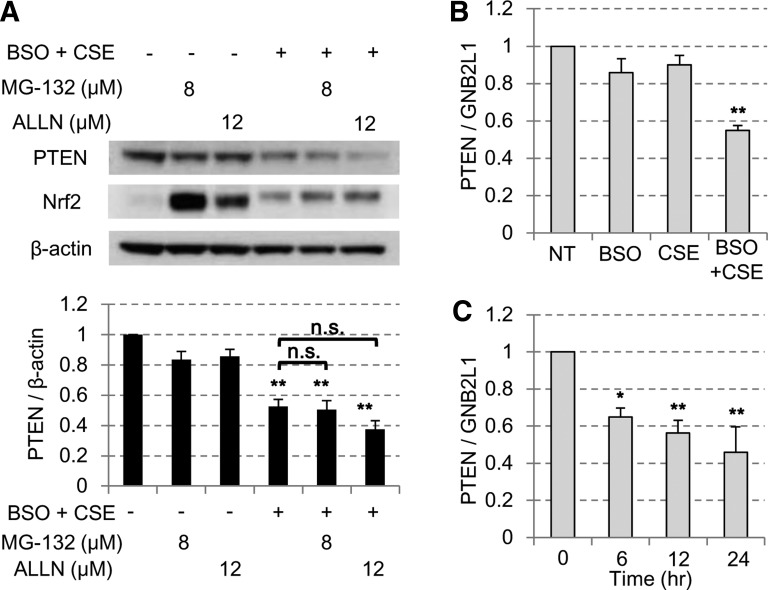
PTEN reduction by CSE was mediated partially through the mRNA suppression. *A*: BEAS-2B cells were treated with or without 3% CSE and 100 μM BSO costimulation in the presence or absence of a proteasome inhibitor, MG-132 or ALLN. PTEN protein levels were then assayed by Western blot analysis. The Nrf2 was used as the standard protein affected by proteasome inhibitors. *B*: BEAS-2B cells were stimulated with or without 3% CSE in the presence or absence of 16 h pretreatment of 100 μM BSO. After 24 h, PTEN mRNA level was examined by reverse transcriptase quantitative polymerase chain reaction (RT-qPCR). *C*: with 3% CSE in the presence of 100 μM BSO pretreatment, PTEN mRNA levels were assayed at each time points by RT-qPCR. All values are mean values ± SE of at least 3 separate experiments. *P < 0.05, **P < 0.01, compared with the values of nontreatment group.

Protein degradation was not responsible for the reduction in PTEN protein levels as the proteasome inhibitors MG-132 (Z-Leu-Leu-Leu-CHO) and ALLN (*N*-acetyl-Leu-Leu-Norleu-al) did not reverse the reduction in PTEN protein levels after the oxidative stress (Fig. 4*A*). By contrast, the mRNA of PTEN decreased significantly but partially in CSE exposure with BSO pretreatment group (Fig. 4*B*), which occurred as early as 6 h after CSE exposure in advance of the PTEN protein reduction (Fig. 4*C*). Therefore, reduced PTEN gene transcription by oxidative stress is partially involved in reducing the PTEN protein levels. As mentioned above, we observed reduction of PTEN mRNA in peripheral lung from GOLD stages 3 and 4 although it was not statistically significant.

#### PTEN-knockdown caused Akt phosphorylation and enhanced cytokine production.

**Fig. 5.**
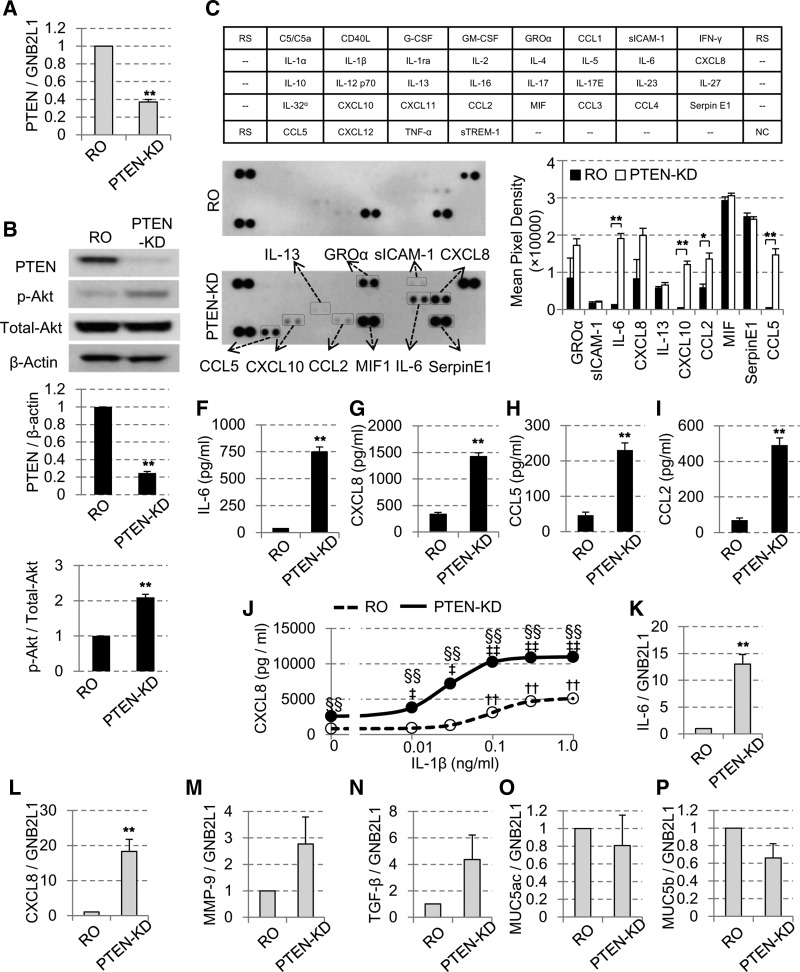
Effect of PTEN-knockdown (KD) on Akt phosphorylation and cytokine production. BEAS-2B cells were incubated with random oligonucleotide (RO; 100 nM) or short interference (si) RNA against PTEN (100 nM) for 48 h. The levels of PTEN were determined by RT-qPCR (*A*) and Western blot analysis (*B*). The ratio of p-Akt/total-Akt as well as PTEN/β-actin were also determined (*n* = 3). Effects of PTEN knockdown on cytokine production determined by cytokine array (*C–E*) or by ELISA for IL-6 (*F*), CXCL8 (*G*), CCL5 (*H*) and CCL2 (*I*). *J*: effect of stimulation with IL-1β (0, 0.01, 0.1, and 1 ng/ml) on CXCL8 release for 24 h in PTEN-KD cells. An impact of PTEN KD on gene expression IL-6 (*K*), CXCL8 (*L*), matrix metalloproteinase-9 (MMP-9; *M*), tranforming growth factor-β (TGF-β; *N*), MUC5AC (*O*) and MUC5B (*P*), all which were corrected with the gene expression of a housekeeping gene GNB2L1 (*n* = 3). All values are mean values ± SE of 3-6 separate experiments. *P < 0.05, **P < 0.01, compared with the values of RO-treated group; **^††^***P* < 0.01, compared with the values of nontreatment RO group; ^‡^*P* < 0.05, ^‡‡^*P* < 0.01, compared with the value of nontreatment PTEN-KD group. ^§§^*P* < 0.01, between the RO group and PTEN-KD group.

To investigate the functional consequence of PTEN reduction, PTEN was knocked down in BEAS-2B cells and several cytokine levels were evaluated. After 48-h incubation with PTEN siRNA (100 nM), we could effectively knockdown both the mRNA (Fig. 5*A*) and the protein levels of PTEN (Fig. 5*B*). The knockdown of PTEN was accompanied with the significant phosphorylation of Akt (p-Akt/total-Akt) (Fig. 5*B*, bottom), and enhanced the secretion of various cytokines, such as IL-6, CXCL8, CXCL10, CCL2, and CCL5 (Fig. 5*C*) in the cytokine array assay, all of which are known to be associated with the proinflammatory response in the pathogenesis of COPD (6). As the cytokine array assay is semiquantitative, ELISA, a more quantitative method was then used, and we confirmed upregulation of IL-6 (Fig. 5*F*), CXCL8 (Fig. 5*G*), CCL5 (Fig. 5*H*), and CCL2 (Fig. 5*I*) after PTEN-knockdown.

As well as basal cytokine production, IL-1β-induced CXCL8 secretion was also significantly potentiated in PTEN-knockdown cells and the dose-response curve shifted leftward (EC_50_ 0.093 ± 0.011 vs. 0.031 ± 0.014, *P* < 0.05; Fig. 5*J*).

The increase in IL-6 and CXCL8 was also confirmed at transcription level (mRNA) by RT-PCR (Fig. 5, *K* and *L*, respectively). In addition, we also confirmed that MMP-9 and TGF-β gene expression was elevated in PTEN knockdown cells, but the gene expression of MUC5AC and MUC5B was not affected (Fig. 5, *M*, *N*, *O*, and *P*).

## DISCUSSION

In the current study, we have shown for the first time that the PTEN protein levels were significantly decreased in the peripheral lung of patients with COPD. PTEN protein levels were positively correlated with the severity of air flow obstruction and showed a strong negative correlation with the Akt phosphorylation, indicating activation of the PI3K signaling pathway. This is consistent with the modulatory role of PTEN on PI3K signaling (41).

The PI3K signaling pathway is an important signal cascade that may be activated by oxidative stress (20), and its prolonged/elevated and inappropriate activation is associated with various pulmonary diseases, such as lung cancer (2, 3, 51), interstitial lung disease (33, 34, 47, 49), and COPD (32, 44, 50). Previously, reports in lung cancer demonstrated a reduction in PTEN as consequence of smoking, but these reports did not differentiate COPD from smokers without airway obstruction. Therefore, it might be possible that PTEN reduction is specific to COPD, even though cigarette smoking is the major risk factor involved in the development of both the COPD and lung cancer (17). The peripheral samples we used were obtained from the tissue bank that collected lung samples from the patients who have made a decision to proceed with lung resection for the treatment of a lung tumor. Therefore, all subjects suffered from low grade or moderate different type of cancer (although noncancerous tissue was used for analysis). This also suggested that the reduction of PTEN is more associated to COPD rather than cancer status. Indeed, reduced PTEN may contribute to the greatly increased risk of lung cancer in COPD patients (1).

Many reports have elucidated the inhibitory effect of the oxidative stress on the PTEN phosphatase activity (23–26, 39, 52), especially via redox regulation of reactive cysteine (Cys124) at the catalytic site of the enzyme. Contrary to our expectations, we could not detect any reductions of IP-PTEN phosphatase activities (normalized by its protein level) in samples from the patients with COPD. In this study, we could collect IP-PTEN protein samples only from lung tissue from limited subjects; therefore, the assay was totally underpowered; in addition, a specific PTEN activity assay system is not available nor are antibodies targeting oxidized PTEN currently available. This potentially limits the measurement of PTEN activity under conditions of oxidative stress. Therefore, it is inconclusive whether the PTEN activity was reduced in COPD or not.

In our BEAS-2B cell model, higher concentrations of CSE (20%) effectively downregulated the expression of PTEN protein (PTEN/β-actin relative ratio vs nontreated control: 0.077 ± 0.028, 92.3% reduction). This was compatible with a previous report that oxidative stress suppresses PTEN expression in human airway epithelial cells (40). However, as the cell toxicity by such a high concentration of CSE was substantial, we could not rule out the possibility of PTEN degradation as a result of cell damage and death. These effects with a higher concentration of CSE might be associated with the acute toxicity of cigarette smoke exposure (33); however, it is difficult to use this model for exploring the pathogenesis of COPD, which is a more chronic reaction to lower concentrations of cigarette smoke. Therefore, we next examined PTEN protein expression in the BSO pretreatment model, which depletes intracellular glutathione storage and may mimic the reduction in glutathione seen in COPD cells. BSO treatment enhances sensitivity of cells to oxidative stress, and therefore, we only needed low concentrations of CSE to avoid any reduction of cell viability seen in high concentrations of CSE. Glutathione concentrations in sputum (46) or muscle (14) are reduced in patients with COPD, and cigarette smoke itself plays a critical role in depleting available glutathione stores in airway epithelial cells (45). Therefore, the combination of CSE exposure with BSO pretreatment may be a more appropriate model of what occurs in vivo in COPD patients. In fact, BSO augmented the effect of CSE in this model, and as low as 3% CSE decreased the protein level of PTEN in a time-dependent manner, without any significant cell injury. This partially explains the different sensitivity against cigarette smoke between the smokers without COPD and patients with COPD as demonstrated by the experiments with primary bronchial epithelial cells from COPD patients (Fig. 1*H*). The replenishment of intracellular glutathione might be more effective than the oral intake of antioxidants (37). In addition, the PTEN reduction by CSE was reversed by *N*-acetyl cysteine treatment, suggesting the involvement of oxidative stress on PTEN reduction (Fig. 3*B*). In fact, we also demonstrated a good negative correlation between the levels of PTEN protein and MDA, a product of lipid oxidation by reactive oxygen species, in peripheral lung tissue (Fig. 1*F*).

Our data show that the PI3K pathway appears to be activated by different mechanisms according to duration of CSE exposure (Fig. 3, *C* and *D*). In the early phase after CSE exposure, Akt was phosphorylated directly by the oxidative stress, without accompanying the PTEN suppression. At later time point, there was a gradual reduction in PTEN, causing prolonged phosphorylation of Akt, even after the disappearance of CSE. These different responses with time suggest that the PTEN is more important for the late-phase reaction to CSE. Also, this might explain why the chronic inflammation progresses even after the cessation of smoking (10) and why some patients may be more prone to acute exacerbations (19a).

We also examined the PTEN-knockdown model using siRNA to reduce PTEN, which resulted in increased p-Akt even under basal conditions and also enhanced the secretion of several proinflammatory cytokines, including IL-6, CXCL8, CXCL10, and CCL5, all of which are increased in the sputum of COPD patients (9, 11). This effect on basal secretion implies a potent basal inhibitory effect of PTEN on normal PI3K signaling. In addition, PTEN-knockdown augmented IL-1β-induced CXCL8 secretion and shifted the dose-response curve of IL-1β stimulation to the left. These results suggested that the PTEN-knockdown cells might be used as the reasonable in vitro model of COPD, with which we can imitate and reproduce the cell responses beyond just CSE exposure. Furthermore, we also confirmed that PTEN regulated gene expression of MMP-9 and TGF-β as well as IL-6 and CXCL8 but did not for MUC5AC and MUC5B; all were involved in pathogenesis of COPD. Thus PTEN controlled expression of specific cytokine or biological factors. Further broad and systematic analysis will be required to clarify the impact of PTEN reduction in all aspect of airway inflammation in future studies.

In conclusion, we have shown that the oxidative stress inhibits the protein levels of PTEN in patients with COPD, resulting in the persistent activation of the PI3K/Akt pathway and resultant proinflammatory mediator release. This may partially explain why COPD progresses even after the cessation of smoking and why some patients are prone to frequent exacerbations. In addition activation of PI3K signaling as a result of decreased PTEN expression may also be important in corticosteroid resistance and accelerated aging as well as the increased risk of lung cancer in COPD (8). Enhancement of the anti-inflammatory PTEN function might be a possible future therapeutic target in preventing COPD progression.

## GRANTS

This project was supported by the Wellcome Trust Programme Grant 093080/Z/10/Z. S. Yanagisawa is a recipient of a Banyu Life Science Foundation International fellowship. P. J. Barnes and C. Vuppusetty are recipients of Wellcome Trust Grant 093080/Z/10/Z.

## DISCLOSURES

P. J. Barnes has served on Scientific Advisory Boards of AstraZeneca, Boehringer-Ingelheim, Chiesi, GlaxoSmithKline, Glenmark, Johnson & Johnson, Napp, Novartis, Takeda, Pfizer, Prosonix, RespiVert, Teva, and Zambon and has received research funding from AstraZeneca, Boehringer-Ingelheim, Chiesi, Novartis, and Takeda. K. Ito is currently an employee of Pulmocide, Ltd., and has honorary contract with Imperial College.

## AUTHOR CONTRIBUTIONS

P.J.B. conceived and designed research. S.Y., J.R.B., C.V., and P.F. performed experiments; S.Y., J.R.B., and C.V. analyzed data; S.Y., J.R.B., C.V., L.E.D., K.I., and P.J.B. interpreted results of experiments; S.Y., J.R.B., C.V., and K.I. prepared figures; S.Y., K.I., and P.J.B. drafted manuscript; S.Y., K.I., and P.J.B. edited and revised manuscript; S.Y., K.I., and P.J.B. approved final version of manuscript.
